# Topics of the Month

**Published:** 1896-11

**Authors:** 


### TOPICS OF THE MONTH.

Concerning the lowering death-rate at Buffalo, the *Journal of the American Medical Association*, September 12, 1896, prints an editorial highly complimentary to the present administration of the health department of this city. Although the statement it makes may impress the casual observer as somewhat remarkable, it is not an exaggeration of existing facts.

The article in question speaks of the death-rate in London, the largest civilised city in the world, as having been reduced to about 17, while in the United States with less concentration of population in large cities there has been less careful administration of health laws, hence a much higher death-rate. It must not be forgotten, however, in this relation that we are fast approaching a similar concentration of urban population as that existing in Europe. Nearly one-fourth of our total population is concentrated in cities of 10,000 or more inhabitants. The increase of percentage in our population is greater in cities than in rural regions. Our aggregate increase is about 7 per cent. a year for the whole United States, 4 per cent. of which goes to the cities.

The importance, therefore, of a rigid administration of the best sanitary laws science can afford is apparent in the light of the foregoing statement. The journal referred to says that it has lately come to its notice that the death-rate in Buffalo has been reduced to 11.67. This is attributable, it asseverates, to the persistent and heroic labors of Dr. Ernest Wende, Health Commissioner, under whose administration Buffalo has reached the lowest death-rate made by any city of the world of equal size. If the saving of one life, continues the *Journal*, is worthy of medals and public honors, what kind of medals and honors should be awarded to Dr. Wende for the thousands that are living in Buffalo today who under a less thorough sanitary management would have died ?

The *Journal* awaits with some interest the answer that Buffalo through her constituted authorities will make to this question in filling the office of health commissioner when the present term expires, December 31, 1896. We confess that it is difficult for us to see but one logical answer, and that is to appoint Dr. Wende to succeed himself.

---

WHILE on the subject of public health we are pleased to note that there is a disposition manifesting in many American cities to discuss, in popular assemblies, problems relating to improvement in sanitation. Lately, according to the *San Francisco Examiner* of October 17, 1896, Rabbi Voorsanger, a famous pulpit orator, in a course of winter lectures on timely topics, delivered an address on the great need of hygienic self-defense. He handled the subject from the practical standpoint of a sound business man coupled with the education of a scholar. He asserted that health, sanitation and hygiene are subjects that he hoped some day would be deemed as important as the three R's in the curriculum of public education ; that the secret of public health is the knowledge of every individual to take care of himself ; that we should avail ourselves of all intelligent means to live long, and that after the commonwealth understands the means of attaining the prolongation of the life of the citizen, he may bow to a community that proceeds intelligently in the path of public duty, which must result in lasting happiness.

The eloquent speaker also affirmed that we need to learn the art of hygienic self-defense; and that we commit suicide through our ignorance of the defined laws of cleanliness, which latter means clean pure homes, clean pure persons, as well as healthy minds. It means also pure, unadulterated food, unpolluted air, pure water, and an intelligent effort to prevent the contamination of these three essentials of good health.

The intelligent presentation of this subject to a popular audience by a man so full of information as Rabbi Voorsanger is to be commended.

---

THE semicentennial anniversary of the discovery of anesthesia was appropriately celebrated at the Massachusetts General Hospital, Boston, Friday, October 16, 1896. The old amphitheater of the hospital had been restored as nearly as could be done to its condition October 16, 1846, at the first public demonstration of anesthesia in surgery.

Distinguished guests were invited to celebrate the event in interesting addresses in prose and verse. Among these were Drs. Robert T. Davis, of Fall River, Mass., who witnessed the original event and who gave an appropriate address of reminiscences; Dr. John Ashurst, Jr., of Philadelphia, who spoke of surgery before the days of anesthesia; Dr. Charles McBurney, of New York, who discoursed upon the surgery of the future, and Dr. S. Weir Mitchell, of Philadelphia, who read a poem on the birth and death of pain. Besides these, Dr. David W. Cheever, of Boston, spoke under the title, What has anesthesia done for surgery? and Dr. J. P. Reynolds, of Boston, delivered an address on Anesthesia in obstetrics. Among those present were Lord and Lady Playfair, of London. Lord Playfair delivered a brief extemporaneous address, in which he gave full credit to the United States for the discovery of anesthesia.

Most appropriately among those present as invited guests were Mrs. William T. G. Morton, widow of the discoverer; Dr. William J. Morton, of New York, his son; Mrs. Frederick Young, of Newark, N. J., a daughter, and Sydney Otis, a grandson, who is now a sophomore at Harvard. The *Boston Medical and Surgical Journal*, in its issues for October 15 and 22, 1896, published the addresses and ceremonies in full, including that of Mr. Charles H.

Dalton, president of the hospital, who welcomed the guests in appropriate words. Our contemporary also published a number of illustrations relating to the event commemorated, and we are indebted to it for the courteous loan of the plate showing the restoration of the old amphitheater that appears on another page of this edition.

---

IN REFERRING to the great event which just now is absorbing so much attention and which has been so elaborately commemorated in Boston, we deem it proper that some mention should be made of the patient who voluntarily took the anesthetic and upon whom Dr. J. C. Warren operated October 16, 1846. His name was Gilbert Abbott, a single man and a printer by trade, and who was possessed of a tumor that discolored the whole of one side of his neck in front—*nevus maternus*—commonly called a birth-mark. The tumor extended along the lower jaw to the maxillary gland and into the mouth, embracing the margin of the tongue. The operation was successful ; at its conclusion the patient declared he had not suffered, and Dr. Warren, the operator, enthusiastically announced that the conquest of pain had been achieved. Whoever may have taken ether before, it remains a fact that to this man it was administered for the first time to make an important surgical operation, and it is he who is represented as the patient in the engraving published elsewhere. We think, therefore, the thanks of the professional world is due to Dr. Washington Ayer, of San Francisco, Cal., an eye witness of this operation, and to Dr. John Homans, of Boston, for calling attention to the propriety of including Abbott's name among those entitled to credit for contributing to the successful application of an anesthetic for the first time in surgery.

